# Optimization Analysis of Two-Factor Continuous Variable between Thread Depth and Pitch of Microimplant under Toque Force

**DOI:** 10.1155/2022/2119534

**Published:** 2022-06-20

**Authors:** Yushan Ye, Jiuyang Jiao, Song Fan, Jieying He, Yamei Wang, Qinghe Yao, Wei Wang, Jinsong Li, Shaohai Chang

**Affiliations:** ^1^Department of Stomatology, Sun Yat-sen Memorial Hospital of Sun Yat-sen University, Guangzhou 510000, China; ^2^Department of Oral and Maxillofacial Surgery, Sun Yat-sen Memorial Hospital of Sun Yat-sen University, Guangzhou 510000, China; ^3^Department of Stomatology, Kaiping Central Hospital, Kaiping 529300, China; ^4^School of Engineering, Sun Yat-sen University, Guangzhou 510000, China; ^5^Urumqi DW Innovation Info Tech Co., Ltd., Urumqi 830000, China

## Abstract

Microimplant, an anchorage device, is widely applied in clinical orthodontic treatment. Since tooth torque is required to be controlled during orthodontic tooth movement, a novel microimplant needs to be developed to apply better torque force during orthodontic. In this study, the optimal value ranges of thread depth and pitch under toque force were studied for choosing microimplant with relevant value ranges in clinical design from biomechanical perspective. Finite element analysis (FEA) and optimization design technology were used for accessing the optimal value ranges of thread depth and pitch under toque force. Thread depth (*D*) (0.1 mm to 0.4 mm) and pitch (*P*) (0.4 mm to 1 mm) were used as continuous variables, with the other parameters as constant, and the optimal value ranges were obtained by analyzing the tangent slope and sensitivity of the response curve. When a torque force of 6 Nmm was applied on the microimplant, the maximum equivalent stress (Max EQV) of cortical bone and maximum displacements (Max DM) of microimplant were analysis indexes. When 0.55 mm ≤ *P* ≤ 1 mm, the Max EQV of cortical bone was relatively smaller with less variation range. When 0.1 mm ≤ *D* ≤ 0.35 mm, the Max DM of microimplant was relatively smaller with less variation range. So in conclusion, the initial stability of microimplants with pitch 0.55 mm ≤ *P* ≤ 1 mm and thread depth 0.1 mm ≤ *D* ≤ 0.35 mm was better with the torque force applied.

## 1. Introduction

Using microimplant as an anchorage in orthodontic treatment has been widely accepted globally among orthodontists [1, 2]. Previous research studies showed that in most of the orthodontic anchorages, the success rate of microimplant anchorage was found to be between 74% and 93%, which was lower than that of regular dental implants reported [2–4]. Microimplant, as a temporary anchorage auxiliary device used widely in orthodontic treatment, exhibited stability from the mechanical embedment of the microimplant and cortical bone [5–7] rather than from the dental implant osseointegration. Therefore, microimplant can be used as orthodontic anchorage right after implantation, due to its efficiency. The stability of the immediate loading after implantation is termed as initial stability, which plays a crucial role for the successful anchorage in orthodontic treatment, as most of the microimplants tend to fail in this period [8].

Many orthodontists reported that the initial stability of implant after implantation can be influenced by many factors, such as the microimplant's shape, diameter, head length, the thread's size, height, and pitch, which are all known to create the stress of the surrounding cortical bone [9–11].

FEA (finite element analysis) is a highly efficient calculating method, mainly used in analyzing static, dynamic objects and physical systems, which can also be used in the study of internal micromotion of objects. Mathur et al. reported the outcome of the implant and its surrounding bone structure's orthodontic load simulated by 3D FEA [12]. Based on 3D FEA, the stress generated was analyzed and the changes in the implant's surrounding bone under orthodontic load could be identified. Similarly, FEA was applied in learning the design parameters of microimplant, such as diameter, length, shape, and size [10, 13–17]. Shen et al. [11] applied a 2 N of mesial-distal horizontal direction load, which was parallel to the maxillary buccal surface, to simulate the horizontal retraction of the anterior teeth in clinical practice. Based on FEA, it was observed that the stress in maxillary and the stability of the microimplant were easily affected by the thread pitch and height, and an increase of 1.20 mm on the thread height proved best at the maxillary posterior zone. Another study showed that the thread size of the microimplant holds zero effects on the stress distribution of the surrounding cortical bone and then its initial stability, based on the application of 2 N linear horizontal force on microimplant using 3D FEM [9].

However, these research studies mainly focused on the linear forces including horizontal traction and vertical force applied on microimplants. In orthodontic treatments, the 3D movements of teeth could not be seen without the torque control of teeth, such as upright of the lingual or buccal inclined molars and buccal or lingual controlling movement of the anterior tooth root [18]. A traditional method for the torque control expects many other teeth containing brackets and steel wire as an anchorage. As reaction force is generated on the anchorage teeth, it makes the treatment more complicated and increases the possibility of adverse reactions like alveolar bone fenestrations and root exposures [19, 20]. Torque control of the tooth determines the controlling root movement of the orthodontics, which can directly affect the outcome of the orthodontic treatment. In order to simplify the root torque control in orthodontic treatment and avoid the adverse reaction, using a microimplant as a torque control anchorage will provide a better outcome [21]. Therefore, a microimplant design specially for torque force controlling anchorage is highly essential. However, the influence of the loading torque on the initial stability of the microimplant is not clearly understood. Using thread depth and pitch as continuous variables, this study probes into the effects of these two variables on bone stress and initial stability of the microimplant, to obtain the optimal value ranges of the two variables under torque force.

Based on the aspects of biomechanics, this study probes further into the thread design requirements for the novel micro-implant and provides a theoretical basis for selecting micro-implants with suitable parameters, which can also meet the clinical requirements of torque force. By improving the microimplant's thread design, its initial stability under the torque force can be easily optimized. Based on our previous research, we have proposed the application of this new type of micro-implant and the design requirements on length, diameter, and thread shape [15, 22–26]. Here, a further study was done on the thread depth and pitch design requirements of the new microimplant for the torque force during the dental treatment, which could promote the initial stability of the microimplant under the torque force.

## 2. Materials and Method

### 2.1. Experimental Model

**Figure 1:**
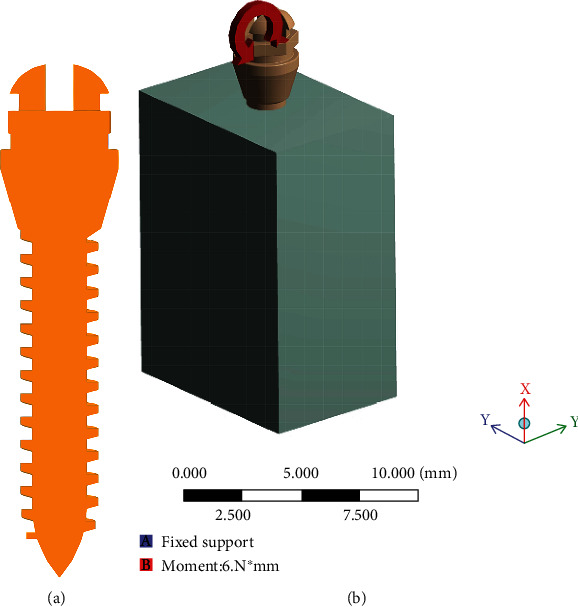
Construction of the model: (a) the model of micro-implant; (b) a torque load of 6 Nmm was applied to micro-implant head.

In the establishment of a 3D finite element model, the basic structural parameters of the microimplant are as follows: length 9 mm, diameter 2 mm, pitch 0.6 mm, and thread depth 0.3 mm. Part drawing stretching command in Pro/E 2.0 was used to draw the cortical bone and cancellous bone. The size of the bone structurally is defined as height 14 mm, length 10 mm, and width 7 mm, where the upper part was the cortical bone with thickness 1 mm, while the lower part was a cancellous bone layer. The basic shape of the thread in the microimplant was the buttress thread with apex angle of 15°. The depth (*D*) and pitch (*P*) were set as continuous variables: the value range of *D* was 0.1 mm to 0.4 mm and that of *P* was 0.4 mm to 1 mm. The microimplant was implanted from the cortical bone into the cancellous bone, in the direction of 90° to the cortical bone surface. The microimplant and the cortical bone were embedded with each other mechanically, and the microimplant was frictionally contacted with bones with a friction coefficient of 0.3 [22]. The above-mentioned model and the microimplant data were imported into the FEA software ANSYS Workbench 13.0 and processed (Figure 1).

### 2.2. Boundary Constraints and Loading Conditions

The boundary constraints indicate that the degrees of freedom of all the nodes in bone's mesial-distal surfaces equates to 0. A torque force of 6 Nmm was applied in the transverse groove at the head of the microimplant in our finite element model, based on Hohmann et al.'s study as they indicated that the torque load greater than 6 Nmm will lead to molar root resorption [23] (Figure 1(b)).

### 2.3. Parameters of the Mechanics of Materials

As per the setting conditions of our previous experiments, bones are the class III bone from Lekholm and Zarb classification, and all the materials were homogeneous, isotropic, and linear elastic materials [24–29]. The biomechanical parameters of the material, including Young's modulus and Poisson's ratio of cortical bone, cancellous bone, and miniscrew, are presented in Table 1. They were set in previous studies, and they are the reference for our study [24–26, 28–30].

### 2.4. Experimental Hypotheses and Definition of the Interface Contact

#### 2.4.1. Hypothesis of Isotropy

The elasticities of an object will be similar in all directions, and the elastic constant will not change with direction.

#### 2.4.2. Hypothesis of Homogeneous Continuity

The object will be in a continuous status, and the whole space of the object will be filled with the media that makes up the object without any space. And the mechanical properties of the object will be similar everywhere. The material that makes up the object fills up the space of the whole object without leaving any space.

#### 2.4.3. Hypothesis of Linear Elasticity

The object will be perfectly elastic. By removing the external force causing deformation, the object still could completely restore to its original shape without any residual deformation. Deformation level will be proportional to stress. Elastic constant does not change with deformation and stress.

#### 2.4.4. Hypothesis of Small Deformation

Slight deformation will occur after the object is loaded, which is called elastic deformation. Removal of the load can restore its original shape.

### 2.5. Analysis Indices

Max EQV was used in cortical bone and Max DM in microimplant as the analysis indices. Thread depth (*D*) and pitch (*P*) were used for the sensitivity analysis of Max DM and Max EQV. The Max EQV in cortical bone was used to represent the deformation resistance of the bone under torque load. The smaller the Max EQV index, the smaller the stress that the surrounding tissue received; then, the possibility of cortical bone being damaged tends to be lesser [26]. Max DM indicates the micromovement of the microimplant under stress, representing the mobility of the microimplant. The smaller the Max DM index, the lesser the mobility of the microimplant [29].

## 3. Results

**Figure 2:**
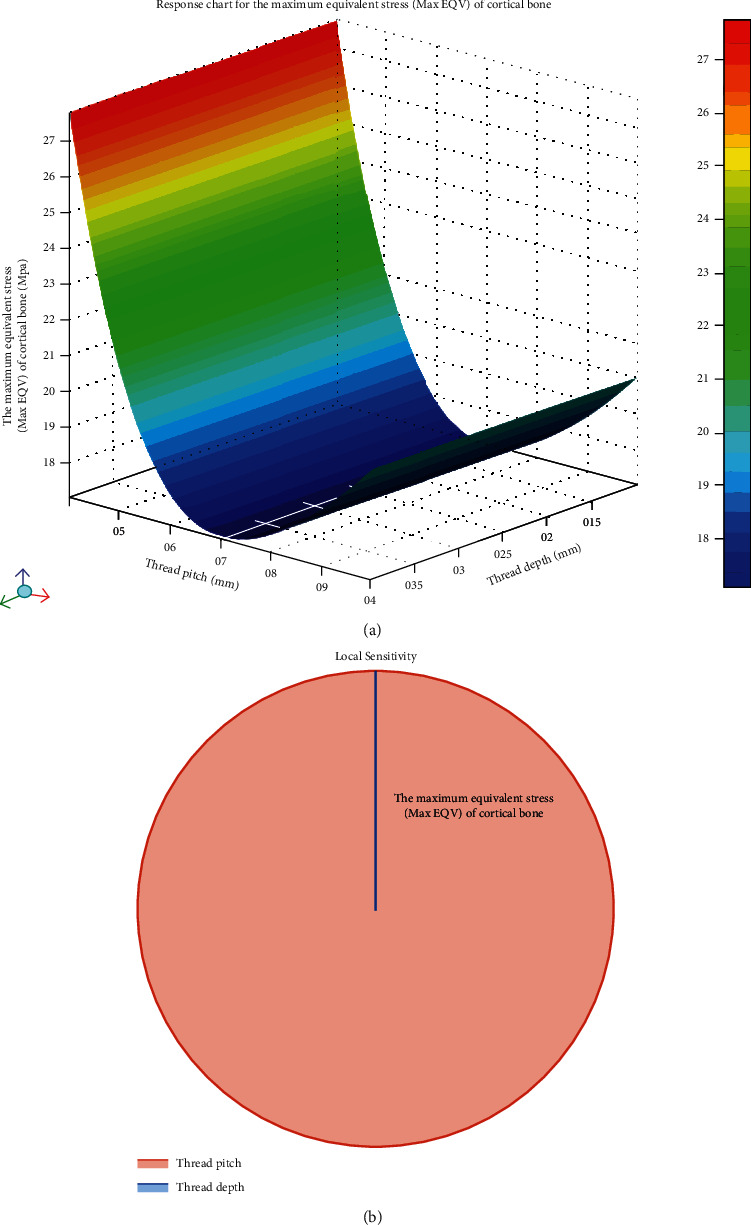
The effects of the continuous changes of *D* and *P* on the Max EQV in cortical bone can be seen in the response surface cloud chart and sensitivity analysis pie chart. (a) With the increase in *P* within the range (*P*: 0.4 mm-1.0 mm), the Max EQV in cortical bone declined by 38.61%; it decreased initially followed by a small increase, but the level in which it was decreased tends to be larger than the increasing range. As *D* increased within the range (*D*: 0.1 mm-0.4 mm), no variation in the values of the Max EQV. (b) The sensitivity analysis of Max EQV in cortical bone. Pitch was sensitive to the Max EQV compared to the depth.

**Figure 3:**
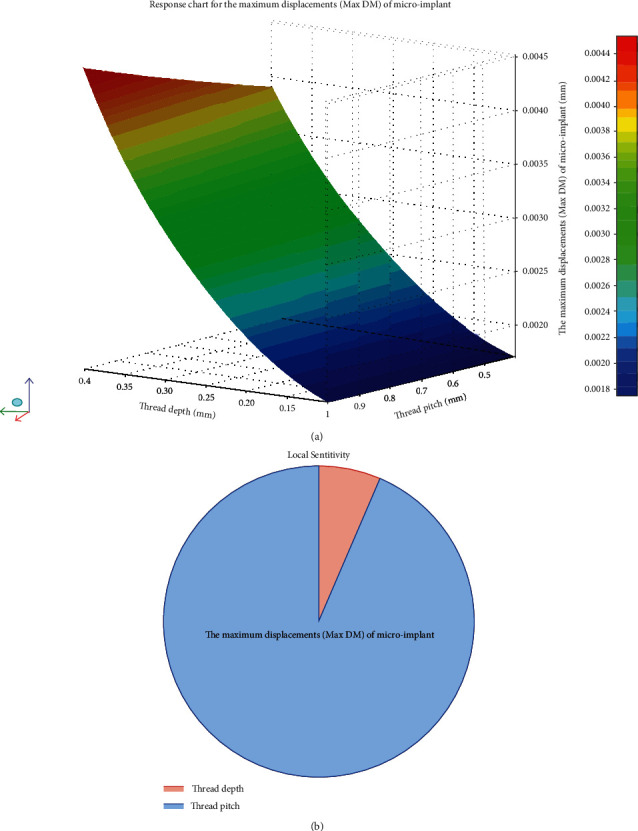
The effects of the continuous changes of *D* and *P* on Max DM in microimplant can be observed in the response surface cloud chart and sensitivity analysis pie chart. (a) With the increase in *P* within the range (*P*: 0.4 mm-1.0 mm), a minimal increase was observed in Max DM. With the increase in *D* within the range (*D*: 0.1 mm-0.4 mm), Max DM increased up to 164.5%. (b) The sensitivity analysis of Max DM in microimplant. Depth was sensitive to the Max DM compared to pitch.

The effects of different parameters of *D* and *P* on the Max EQV in cortical bone and Max DM in microimplant can be observed in the response surface cloud chart and sensitivity analysis pie chart (Figures 2 and 3, respectively), among which, decline range ((Maximum peak − Minimum peak)/Maximum peak × 100%) and increasing range ((Maximum peak − Minimum peak)/Minimum peak × 100%) were expressed. With *P* increasing within the range (*P*: 0.4 mm-1.0 mm), Max DM showed a slight uptrend and Max EQV fell by 38.61%. When *D* increased within the range (*D*: 0.1 mm-0.4 mm), the values of Max EQV were similar and Max DM increased up to 164.5%.

**Figure 4:**
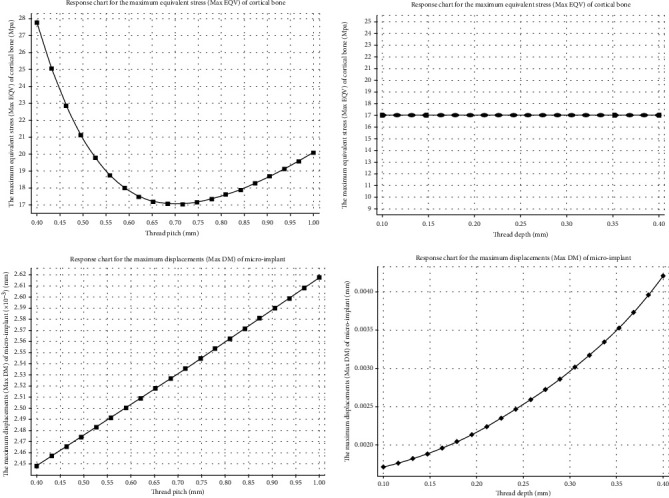
When one factor was set at intermediate value in the range, the effects of another factor on the Max EQV in cortical bone and Max DM in microimplant were evaluated in the response curve.

Our research focused on the variations of two-factor continuous variables *D* and *P*. When one of them was at intermediate values, the response curve to the objective function of the other one is shown in Figure 4. As the thread pitch increased, Max EQV fell by 62.88%. Max DM increased slightly (6.91%). The increase in thread depth influenced the Max DM with rapid increase (144.45%).

**Figure 5:**
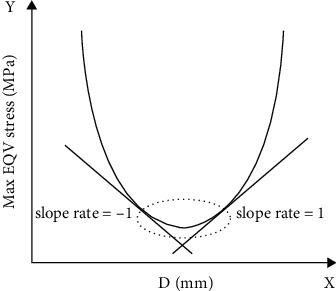
The optimum selection of the curve can be observed in this chart of slight changing and minimal value of the curve.

The determination method of the optimal value ranges of the response curve indicates that when a straight line was tangent to a curve, the slope of the straight line reflects the changing rate of the curve (Figure 5). When the slope was between -1 and 1, it showed the objective function changes by the variables in a mild manner. Similarly, if the value of the objective function was relatively small while the slope was within the range, the optimal variable parameters should be selected from this range [30]. When 0.55 mm ≤ *P* ≤ 1 mm, the Max EQV of cortical bone was relatively smaller. When 0.1 mm ≤ *D* ≤ 0.35 mm, the Max DM of microimplant was relatively smaller.

## 4. Discussion

### 4.1. Optimization Design and Analysis

A multiobjective optimization analysis (DesignXplorer) module is an optimization design module integrated in ANSYS Workbench for a collaborative design optimization environment [23]. It achieves the optimization scheme by advanced sampling technology. The principle of using the Monte Carlo method was collecting design parameter samples followed by calculating the response results of each sample, and by using a quadratic interpolation function, the response surface cloud chart was constructed, as well as the response curve of the design space. In this study, we sampled the required parameters of the model by ANSYS Workbench13.0 and established the response surface and response curve. The parameters were imported into Unigraphics through the two-way seamless connection between ANSYS Workbench and Unigraphics. After obtaining the entity model, it was imported back to ANSYS Workbench through the connection. By finite element calculation, the parameters were identified after comprehensive sampling and analyzed the Max EQV and Max DM, respectively. The response surface represents the 3D response chart cooperatively affected by the two factors on the evaluation index. And the response curve represents the 2D response chart when one factor was the intermediate value, while the other one works on the evaluation index of the model. Sensitive analysis was used to measure the effects of each factor on the result of the system or model to judge which factors might affect more.

Many scholars have reported the research on the mutual influences of different configuration factors between implant and microimplant by continuous bivariate analysis. Kong et al. [30] evaluated the mutual influence of the implant's length and diameter and their optimal value ranges using bivariate analysis. Reynders et al. [8] also applied bivariate analysis to identify the optimal value ranges of thread height and pitch. Their research was different from our study as they focused on horizontal linear force, while our objective was on torque force. We have earlier reported the research on using bivariate analysis for the optimization design of a microimplant with the length and diameter changing continuously under different forces, and it provided a theoretical basis for the selection of microimplants with optimal sizes in clinical treatments [33]. The thread depth and pitch work cooperate effectively on the initial stability of the microimplant. They are interconnected on the initial stability of the microimplant. In our study, *D* and *P* act as two-factor continuous variables, and we proposed a bivariate analysis which was closer to the real situations than a single-factor discrete variable in clinical treatments. And the research results provided more direct and accurate data for the design parameters of the microimplant. The application of the proposed microimplant has already been reported in our previous research [15], such as the design requirements of length and diameter [23]. This paper studied further on the thread design requirements to meet the requirements of torque force in orthodontic treatment. Optimizing the design of the thread could increase the initial stability of the microimplant under torque force. The purpose of the optimization and analysis of *D* and *P* was to identify the most optimal *D* and *P*.

### 4.2. Optimization Analysis of Thread Depth and Pitch

The initial stability of microimplant is the core factor for the stable and reliable orthodontic treatment. Several scholars have learnt the effects of its shape and thread design on the initial stability of the microimplant from different aspects using different methods, with a lot of research studies on the reasonable value ranges of thread depth and width [31–33].

An earlier study analyzed the effects of the pitch on the initial stability of the microimplant by measuring the highest insertion torque and pull-out strength of the microimplant and identified that by minimizing the thread pitch, a decrease in pull-out strength was observed. A study based on the pull-out test on four groups of self-drilling microimplants with different geometric design features proposed that the geometric design features does not hold any noticeable effects on the initial stability of microimplants [35]. That is to say, with higher thread pitch, side, thread angle, and top angle, the initial stability of microimplants was higher.

A 3D FEM revealed that by keeping the external diameter of the microimplant unchanged, increasing the thread size (depth and width), and decreasing the internal diameter, there was increase in stress on the cortical bone. When the external diameter was 1.4 mm, the minimum stress on the cortical bone of microimplants with no thread was generated. When the depth was 0.3 mm, the maximum stress was generated. When the thread depth decreased from 0.33 mm to 0.1 mm, the maximum stress on the cortical bone decreased by 61% [9].

Based on 3D FEM and pull-out test, Chang et al. [10] studied the effects of the thread depth on the initial stability of the microimplant under a lateral load of 3 N horizontal force on it when its external diameter was 2 mm, and it was observed that when the ratio of internal and external diameters was 0.68, it generated the maximum pull-out resistance with higher initial stability.

On analyzing the effects of the thread pitch on the microimplant's initial stability based on measuring the maximum screw insertion torque and the pull-out strength, it showed that the optimal pitch tends to be between 0.75 and 0.80 mm [34]. Microimplant with too large or small pitch values was unfavorable for its initial stability. Applying 3D FEM and pull-out test, Sana et al. [36] evaluated the stabilities of three different microimplants based on Thread Shape Factor (TSF) and calculated the average thread depth, pitch, and their relationship and identified that microimplants with relatively bigger diameter, smaller pitch, and shorter taper did have better geometric features and initial stability.

As per the above-mentioned research reports, forces applied were all linear including horizontal traction, retraction, and vertical and lateral forces. But in orthodontic treatment, the movement of teeth cannot be done without torque control. Therefore, it is essential to find out a microimplant suitable for orthodontic treatment under torque force, which could form a good orthodontic anchorage system to avoid reaction forces on other teeth as anchorage, simplify the treatment process with more direct and efficient control on the teeth's torque force, and reduce adverse reactions.

Our study observed the variations of the Max EQV in cortical bone and Max DM in microimplant (Figures 2 and 3) after 6 Nmm torque force applied on the microimplant. With *P* increasing within the range (*P*: 0.4 mm-1.0 mm), the Max EQV in cortical bone showed a thorough downtrend, and Max DM showed a slight uptrend. The values of Max EQV decreased initially and then increased, but the range of decrease was highly significant compared to that of increase. The Max EQV fell by 38.61%. When *D* increased within the range (*D*: 0.1 mm-0.4 mm), the values of Max EQV were similar and the displacement peak of the microimplant was in an uptrend with an increase up to 164.5%. The data certified that the effects of the thread pitch and depth on cortical bone and microimplant micromovement were different. From the sensitivity analysis pie chart, the effects of thread pitch were much greater than those of depth with regard to Max EQV (Figure 2). So, when we want to decrease the cortical bone stress, attention needs to be paid on the thread pitch rather than on depth. As to the microimplant's displacement, the thread depth tends to be more influential than the thread pitch. Therefore, more attention should be paid on the thread depth design rather than on the thread pitch to decrease the microimplant's micromotion.

When one factor was set at intermediate value in the range, we discussed the effects of the other factor on the Max EQV in cortical bone and Max DM in microimplant. We found that the increase in the thread pitch led to a decrease trend on Max EQV. As the thread pitch increased, Max EQV in cortical bone decreased firstly and then increased. The decrease range was larger than the increase range, and as a whole, the Max EQV showed a downtrend trend in an asymmetric parabola. The Max EQV fell by 62.88%. Meanwhile, with the thread pitch increased, the max displacement of the microimplant increased slightly (6.91%) in a straight line. On the other hand, the increase in thread depth had no impact on the Max EQV in cortical bone, while it did influence the Max DM in microimplant in an asymmetric parabola with rapid increase (144.45%).

Through the analysis of the tangent slope of the response curve, it showed that when 0.55 mm ≤ *P* ≤ 1 mm, the values of Max EQV in cortical bone were small with limited changes; when 0.1 mm ≤ *D* ≤ 0.35 mm, the values of Max DM in microimplant were small with limited changes. In total, when the thread pitch of microimplant varies from 0.55 mm to 1 mm and the depth varies from 0.1 mm to 0.35 mm, the optimal initial stability of microimplant could be achieved.

## 5. Conclusions

Setting thread diameter and pitch as the two-factor continuous variables, we drew the response cloud charts and response curves, respectively, using ANSYS Workbench 13.0. The two-factor analysis performances were better and identical to real clinical treatment than the single-factor discrete variable analysis, by providing a more direct and accurate certification for the design parameters of the microimplant.

With the increase in thread pitch, the values of Max EQV in cortical bone decreased initially, followed by a small increase, showing an overall sharp downtrend, while the values of Max DM in microimplant were in a slight upward trend. On the other hand, as thread depth increased, the values of Max EQV in cortical bone remained unchanged, while the values of Max DM in microimplants increased sharply.

Therefore, to reduce the cortical bone stress, the thread pitch is a key important factor. Meanwhile, to reduce the microimplant movement, the thread depth plays a vital role.

In summary, at 0.55 mm ≤ *P* ≤ 1 mm and 0.1 mm ≤ *D* ≤ 0.35 mm, the microimplants achieving optimal initial stability tends to be higher.

## Figures and Tables

**Table 1:** The biomechanical parameters of the material.

	Young's modulus (MPa)	Poisson's ratio
Cortical bone	13,700	0.33
Cancellous bone	1,600	0.3
Micro-implant	110,000	0.35

## Data Availability

All the materials and data have been presented in the main paper.

## Abbreviations

FEA: Finite element analyses
Max EQV: The maximum equivalent stress
Max DM: Maximum displacement of micro-implant
EQV: Equivalent stress
DM: Displacement.
